# Experimental and Numerical Investigation on Pile Foundation Underpinning Structure System in Urban Overpass

**DOI:** 10.3390/ma16196576

**Published:** 2023-10-06

**Authors:** Lei Yan, Xiaoying Gou, Zhengchao Guo, Xin Zhang, Yu Jiang, Xingwen Ran, Guanwen Chen, Kefeng Yue

**Affiliations:** 1 School of Civil Engineering, Chongqing Three Gorges University, Chongqing 404100, China; yanlei1988413@163.com (L.Y.); b195987729@163.com (X.Z.); jiangyu2000213@163.com (Y.J.); stzbwgj@163.com (X.R.); liusongrui1231@163.com (G.C.); 2 School of Civil Engineering, Chongqing University, Chongqing 400045, China; 3 State Key Laboratory of Mountain Bridge and Tunnel Engineering, Chongqing Jiao-Tong University, Chongqing 400074, China; 19123653564@163.com

**Keywords:** pile foundation replacement beam construction, polymer reinforced planting bar adhesive, interface between new and old concrete, experiment, finite element simulation analysis

## Abstract

In view of the complexity of the pile foundation underpinning structure system and the stringent requirements of the construction process, this paper briefly describes the necessity of introducing epoxy resin reinforcing adhesive of planting rebar in the design of pile foundation underpinning beam structure to improve the mechanical properties of the reinforced beam new and old concrete joint surfaces and proposes a new type of pile foundation replacement beam system construction method by “chiseling + prestressed reinforcement + epoxy resin reinforcing adhesive”. This paper uses an actual pile foundation underpinning project of an urban overpass as a prototype, designs and creates a model structure with a similarity ratio of 1/6, and performs repeated progressive static loading tests to study the load carrying capacity, displacement change, and other properties of the strengthened replacement structure, as well as analyses and distorts the overall working performance and failure mode of them. On this basis, the prototype structure’s finite element analysis model was built, and the finite element analysis results were compared with the test results to obtain the mechanical properties and deformation characters of the actual pile foundation underpinning structure system corresponding to the actual underpinning beam load. This paper’s study can lay the theoretical and experimental foundation for the smooth development of similar projects.

## 1. Introduction

With the acceleration of urbanization, urban rapid rail transit systems, represented by subway tunnels, have been extensively constructed. However, due to the rapid growth in the number of urban structures, situations involving the construction of new bridges or subway tunnels near or through existing buildings have become increasingly common. In order to ensure the safety of the upper structures, pile foundation replacement technology is often employed to transfer the superimposed loads from existing piles, ensuring the smooth progress of new construction projects [1,2,3,4,5,6]. Pile cap replacement technology is an effective construction technique that involves the creation of a new replacement beam and replacement piles to efficiently transfer the existing load-bearing system. This process redirects the loads previously borne by the existing piles onto the newly constructed replacement piles, effectively managing the stresses and deformations in the existing upper structure [7,8,9].

As urban underground construction continues to grow rapidly, the significance of pile foundation replacement construction technology for urban development becomes increasingly prominent. The pile foundation underpinning technology was first applied to Winchester Cathedral in the United Kingdom. Divers dig a trench through the peat and chalk layers to access the gravel layer, then fill it with concrete to carry out underpinning construction [10]. Germany has conducted several theoretical and practical investigations into pile foundation underpinning technology since the 1950s, employed this technology in numerous projects, and included pile foundation underpinning technology in its industrial standard (DIN) [11,12]. Shan et al. [13] conducted pile underpinning research on an actual building, and the results indicated that the piles acted well as an underpinning role in transferring structure load into surrounding geomaterials, which may offer enough support for the rebuilt structure. Xu et al. [14,15], based on the engineering background of shield tunnel crossing through group pile foundation of a road bridge with pile underpinning technologies in Shanghai, verified the feasibility of pile underpinning technology to solve this problem. Li et al. [16] investigated the stress transfer mechanism during the underpinning process by conducting the refined numerical simulation of the pile foundation underpinning construction using FLAC3D, and found that pile foundation underpinning technology could effectively transfer the overlying load on the underpinning pile to a new underpinning pile. That confirmed the significance of pile foundation displacement construction technology to urban infrastructure projects.

There are numerous factors that influence the safety of pile foundation replacement beam construction. Employing appropriate construction methods and effective safety control measures are essential safeguards for pile foundation replacement beam construction technology. In this regard, scholars, both domestically and internationally, have conducted extensive research. Li et al. [17], Dong et al. [18], Wang [19], and Huang et al. [20] investigated the mechanical performance of replacement piles under replacement loads and proposed a method for predicting the pile’s bearing capacity. Huang Xi et al. [21], by analyzing the transfer of replacement loads, the settlement patterns of pile foundations when a tunnel traverses the pile foundation of a flyover, and the impact of design parameters on the safety of pile foundation replacement construction, have found that active replacement is superior to passive replacement. Furthermore, they observed a positive correlation between the pile length, pile diameter, replacement beam height, and the safety of the pile underpinning replacement structure. Using the orthogonal experiment of scaled models of the wrap-underpinning joints under frame columns in moving engineering, Zhang et al. [22] investigated the influences of the shear-span ratio, longitudinal reinforcement parameter, and stirrup ratio in the underpinning joint surface on the orthogonal experiment of scaled models of the wrap-underpinning joints under frame columns. They also provided the formula to calculate the bearing capacity of underpinning joints. Xu et al. [15] conducted a seismic analysis of pile foundation replacement structures to elucidate the load transfer mechanism of bridge structures throughout the entire construction process. Meanwhile, Zhang et al. [23] established a numerical simulation model that accounted for the entire foundation replacement structure and construction steps to validate the feasibility of the pile foundation replacement construction scheme. Yue et al. [24] tested sixteen prototypes considering different shear span-to-depth ratios, underpinning joint heights, and reinforcement ratios and discovered that the underpinning beams could fail in shear or flexure-shear. During loading, the interface between the column and the beam would also be prone to failure. Yan et al. [25] studied the shear and slide characteristics of the joint and developed a revised method for determining shear capacity using three local node models of the underpinning structures with a similarity ratio of 1/1. Horpibulsuk et al. [26], Wang et al. [27], Kou. [28], Tu et al. [29], and Igba U.T. et al. [30] proposed an optimized pile cap replacement construction scheme based on practical engineering applications. The aforementioned research has elucidated the key factors affecting pile foundation replacement beam construction technology, including pile foundations, replacement beams, and replacement piles. It has also proposed relevant computational models, laying a solid foundation for the practical application of pile foundation replacement beam systems and paving the way for further exploration of novel pile foundation replacement techniques.

However, there is a notable lack of research addressing the issue of the connection between newly cast replacement beams and existing structures in pile foundation replacement beam construction technology, specifically, the adhesive properties at the interface between old and new concrete. Bond slippage between the new and old concrete contact surfaces of pile foundation underpinning beam structures has become the key to limiting the use of underpinning construction technology in large-scale projects due to insufficient anchoring technology and bond material limitations. Fortunately, the development of polymer composite reinforcing adhesive anchoring technology for pile foundation underpinning has created new ideas. Epoxy resin reinforcing adhesive of planting rebar as a commonly used composite building structural adhesive material, with excellent adhesive properties, chemical stability, and strong heat resistance, has attracted scholars in various fields of research [31,32] while also adding a variety of new polymers of composite epoxy resin materials and new epoxy resins with specific properties have also appeared ones after another, such as the novel in situ polytriazolesulfone modified epoxy and the epoxy resin modified with biodegradable polymer [33,34,35]. De Maio et al. [36] proposed the reinforcing effect of nano-modified epoxy resin on the structural response of fiber-reinforced polymer (FRP)-plated reinforced concrete (RC) components and emphasized the beneficial effects of the nano-enhanced epoxy resin on the bond strength between concrete and FRP systems. Wang et al. [37] found that the addition of silica particles could obtain an epoxy adhesive with balanced stiffness-toughness by dynamic mechanical analysis and thermogravimetric analysis. Albahkali et al. [38] demonstrated the concentration of Al_2_O_3_ nanoparticles and graphite increased addition is positively correlated with the performance of the epoxy nanocomposites using the frictional tests. Michałowska-Maziejuk et al. [39] applied carbon fiber strips boned into the concrete reinforcement cover to strengthen the preloaded reinforced concrete beams and confirmed that heating the strip could accelerate the strengthening process by experiment. Liu et al. [40] pointed out that cracks in building structure adhesives can be self-healed in various ways and proposed a new epoxy with self-repairing properties for environmentally friendly building structure adhesives, which is helpful to the prolongation of the reinforcing adhesive of planting rebar’s working life in complex climatic and engineering environments and also opens the door to the future application of a new type of the reinforcing adhesive of planting rebar to pile foundation underpinning technology. He et al. [41] conducted a study on the mechanical properties of a new epoxy resin reinforcing adhesive for planting reinforcement in highway engineering and discovered that the use of a new composite material can not only greatly improve bond strength between structures but also greatly improve over-all structural load-bearing performance. Gou et al. [6] proposed five different beam-column joint forms and demonstrated that the combination of “tongue and groove + anchor bar + prestressed force” can effectively guarantee the safety of beam-column joints. That provides a valuable reference for the combination of the new type of epoxy resin reinforcing adhesive and reinforcing bar construction technology. It can be seen that the epoxy resin reinforcing adhesive composite material has the high bonding characteristics that traditional planting reinforcement construction requires. Now, the epoxy resin composite material has been widely used in various fields with a good research foundation and technical reserve, which is beneficial to the realization of pile foundation underpinning technology in the complex project, and it is worth introducing into the attempts. However, aiming at reinforcing adhesive excel-lent material performance, it must be combined with reasonable planting reinforcement construction technology, such as the rough interface—prestressing—planting rebar—reinforcing adhesive construction technology methods, such as joints with a rough interface + planting reinforcement + prestressing + epoxy resin reinforcing adhesive of planting rebar connections.

In summary, researchers have conducted more practical engineering applications for pile foundation underpinning technology; however, the safety of “chiseling—prestressed reinforcement—epoxy resin bonding” for treating the interface between old and new concrete has yet to be thoroughly tested and studied. Based on this, the paper uses an actual pile foundation underpinning project of an urban overpass as a prototype, employs a method involving chiseling—prestressed reinforcement—epoxy resin bonding simultaneously designs and creates a model structure with a similarity ratio of 1/6, and analyses and discusses the overall working performance and failure mode of them. We expect that this paper’s study can lay the theoretical and experimental foundation for the smooth development of similar projects. It also lays the foundation for the future development of the deep integration between the novel epoxy resin reinforcing adhesive and the great planting rebar technology.

## 2. Design of the Testing

### 2.1. Similarity Relation

The geometric similarity ratio of the model was chosen as 1/6 when combined with the test demand and test circumstances. Other model similarity constants were inferred using fundamental mechanics equations or the approach of comparable dimensional analysis, as indicated in Table 1.

### 2.2. Model Design and Construction

**Figure 1:**
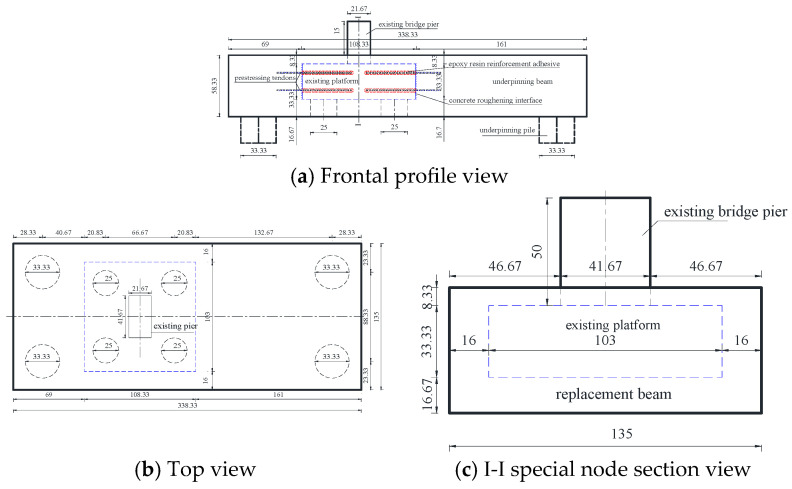
Model design drawing (cm).

**Figure 2:**
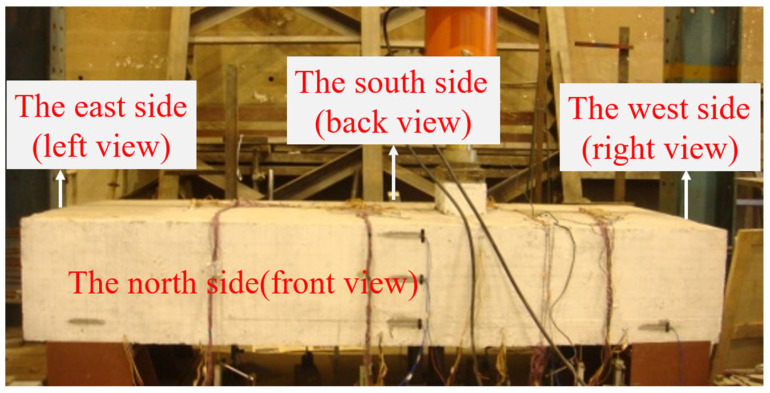
Model physical drawing.

The similarity ratio was used to determine the dimensional correspondence between the model and prototype structures, as shown in Table 2 below. Figure 1 illustrates the intended model structure. The joints with rough interface, planting reinforcement, prestressing, and construction adhesive were applied to manufacture the model depicted in Figure 2 according to the structure design drawing.

### 2.3. Model Materials

The materials used for the underpinning beam model are the same as those used for the prototype construction. The concrete strength grade is C50, and the major reinforcement in the compression and tension zones of the underpinning beam is HRB400, while the other bars are HRB335 reinforcement. The underpinning beam’s longitudinal prestressed tendons are made of high-strength, low-relaxation steel strands with a standard tensile strength of *f_pk_* = 1860 Mpa and a nominal diameter of 15.2 mm. The results of material tests are displayed in the Table 3 below.

The novel planting reinforcement anchoring method, “joints with rough interface + planting reinforcement + prestressing + epoxy resin reinforcing adhesive of planting”, is used for the interface between old and new concrete of the underpinning beam. Epoxy resin as a planting reinforcement of thermosetting Resins, which has excellent mechanical properties, adhesion, and chemical stability, as well as low material shrinkage and a low cost of preparation, and is widely used in construction, adhesives, composites, and other fields. Therefore, the planting reinforcement anchoring technique employs epoxy resin glue as the adhesive of planting reinforcement and concrete. The material properties of epoxy resin are shown in Table 4.

**Figure 3:**
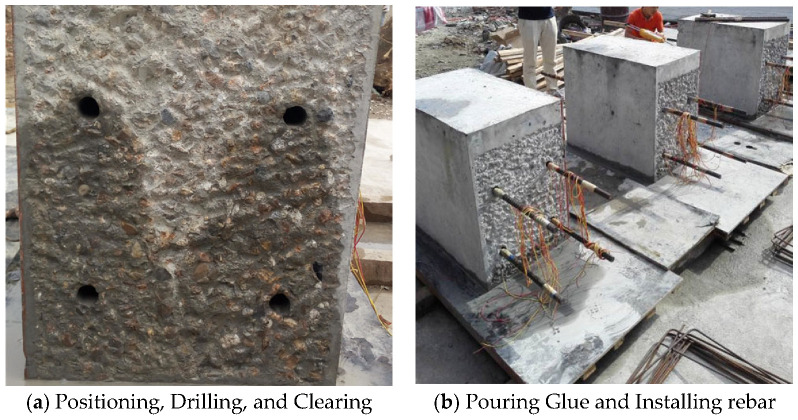
Construction process of planting reinforcement.

The following is the construction process for planting reinforcement between the new pouring concrete and the existing concrete interface for the pile foundation underpinning beam structure: measuring the holes for planting rebar installation, drilling, and cleaning holes, filling the epoxy resin anchoring adhesive of planting reinforcement, installing the planting rebar and maintaining, as shown in Figure 3.

### 2.4. Test Setup and Measuring Point

The geometric similarity ratio for the experimental model in this study is 1:6. The testing equipment used is a computer-controlled electro-hydraulic servo shear testing machine. The test loading system consists of a 5000 kN hydraulic jack and a roller system. The loads are precisely measured using pressure sensors and collected and recorded using a data acquisition system. Throughout the entire loading process, a repeated progressive static loading method is employed, which includes loading, unloading, and then reloading. Before applying a replacement load to the specimen, it is preloaded twice with a load of 100 kN. Subsequently, loading is carried out in stages, with 150 kN applied as the first stage, repeatedly until reaching 1850 kN. Finally, the load is incrementally increased by 100kN stages until the specimen reaches its ultimate load capacity. To ensure load and deformation values stabilize, a two-minute holding period follows the completion of each stage of loading. The loading protocol used in the experiments is displayed in Table 5.

**Figure 4:**
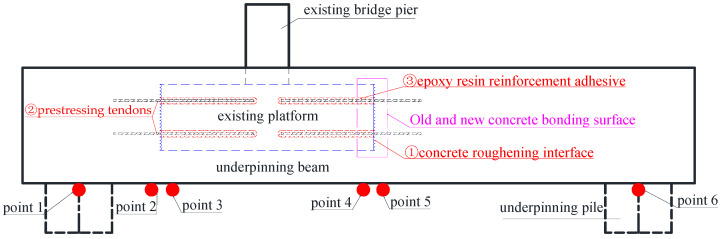
Layout of measuring points.

The test in this work is primarily intended to analyze the displacement response of the drag-replacement structure under load, and six displacement gauges are set, as shown in Figure 4 below. Simultaneously, Figure 4 is employed to vividly present the novel anchoring connection method with (1) chiseling (concrete roughening interface), (2) prestressed reinforcement, and (3) epoxy resin bonding.

## 3. Test Results

### 3.1. Experimental Phenomenon

**Figure 5:**
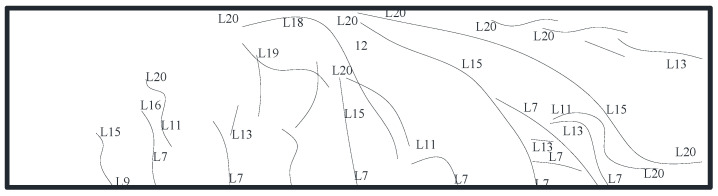
Development of cracks on the south side.

**Figure 6:**
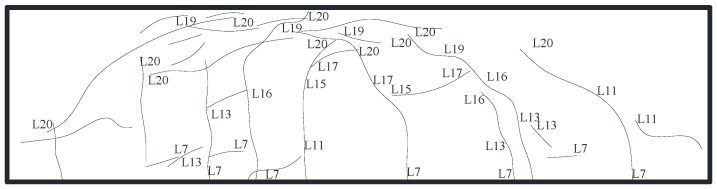
Development of cracks on the north side.

**Figure 7:**
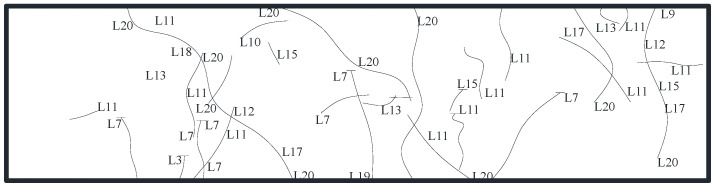
Development of cracks on the bottom surface.

The specimen did not display a noticeable damage phenomenon at the start of loading due to the low loading force. When the load was increased to 550 kN, for the first time, a little fracture occurred at the bottom of the underpinning beam below the loading point, and the specimen was still in the elastic stage. When loaded to 1150 kN, multiple horizontal cracks appeared at the bottom of the beam between 1/2 section on the left side of the bearing platform and 1/2 section on the right side of the bearing platform and gradually developed to both sides of the beam and multiple vertical and inclined cracks appeared on the south and north sides of the beam, with small crack widths. When loaded to 1750 kN, the bottom of the beam appeared along the east–west direction with a number of horizontal newborn cracks, intersected with the original cracks, and gradually formed a network of cracks; the original cracks continued to develop and form a number of north–south penetrating cracks, crack width increased, inclined cracks extended, and the slope gradually decreased; When loaded to 2750 kN, the bottom of the beam formed a number of full-length cracks with a crack width of up to 0.5 mm, and beam side inclined cracks near the loading point intersected. At the same time, the beam was heard to make an abnormal noise, displacement increased significantly, the load value reached its peak and appeared to decline, the maximum crack width reached 0.8 mm, the specimen was considered damaged, and loading was stopped. Figure 5, Figure 6 and Figure 7 depict the specimen’s ultimate cracking state. L represent the loading stage.

### 3.2. Specimen Load Capacity

When the load on the displacement beam ranged from 0 kN to −1900 kN, the behavior of the underpinning beam exhibited linear elastic deformation. As the load continued to increase, a turning point appeared in the load–displacement curve with a decrease in the curve’s slope. When the load reached the maximum design load of 2750 kN during the test, a peak appeared in the curve, indicating that the specimen was approaching failure. The phase from the turning point in the curve to reaching the ultimate load represented the plastic deformation stage of the underpinning beam. Afterward, as the load continued to increase, cracks in the specimen rapidly developed and propagated until failure occurred.

The load–displacement curve for the transfer beam under transfer loads is similar to the deformation curve of the steel reinforcement. This similarity is attributed to the optimized bonding performance of the new planning rebar anchoring technology at the interface between old and new concrete in the underpinning beam. This optimization overcomes the weak points in the underpinning structural system, enhances the deformation performance of concrete, and thus allows the steel reinforcement to play a primary controlling role in the deformation of the underpinning structural system.

**Figure 8:**
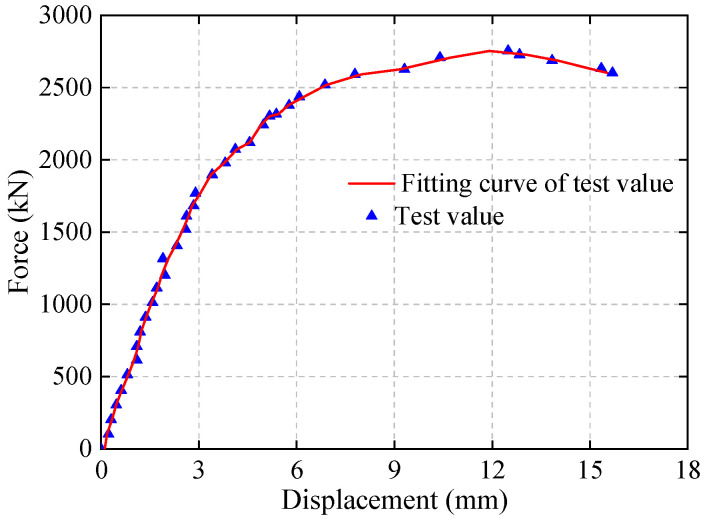
Load displacement Curve.

### 3.3. Specimen Load Capacity

**Figure 9:**
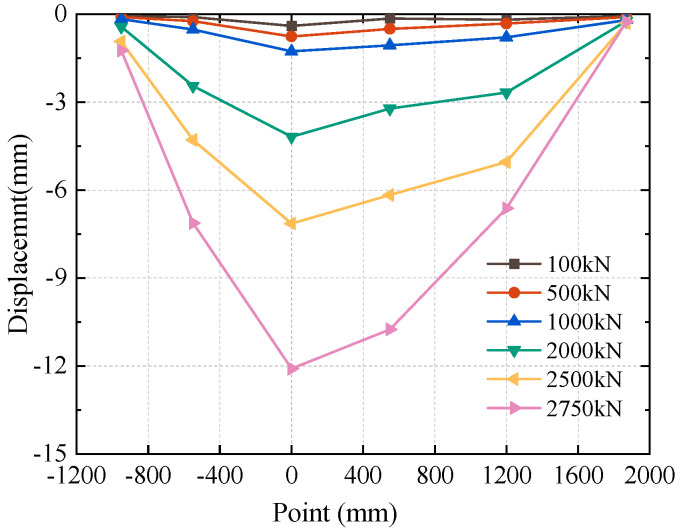
Deformation of the beam body.

The displacements of the underpinning beam at different load values and different measurement places were obtained by extracting the results of the displacement of the bottom of the beam under different load values in the underpinning beam model test, as shown in Figure 9. From this figure, when the load is 1000 kN, the maximum displacement of the lower part of the underpinning beam is 1.27 mm, and the displacement of the original underpinning beam is 7.62 mm, indicating that the displacement of the structure after the completion of the underpinning beam construction is smaller and the overall stiffness is greater under the action of the upper large-axis bridge load. The beam deformation is more coordinated in the elastic range, meaning that the internal concrete old and new bonding surfaces are reliable, and it is absolutely viable to finish the treatment of the underpinning structure’s concrete bonding surface using this approach. The deformation of the abutment region clearly exhibits incoherence once the specimen reaches the yielding stage, indicating that the force in this region of the beam is more concentrated and that it is the weak area of the structural damage.

## 4. Finite Element Simulation Analysis

### 4.1. Model

**Figure 10:**
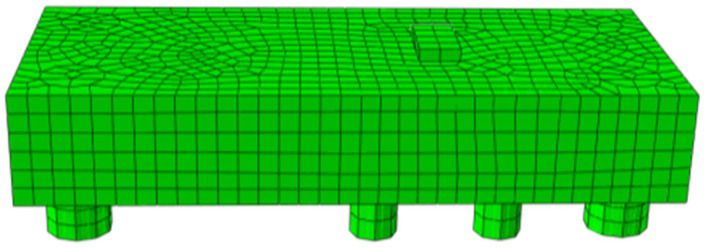
Finite element model of underpinning structure.

The general finite element software Abaqus is used in this research to create a proto-type simulation model of an underpinning beam structure. Firstly, concrete elements are modeled using C3D20R solid elements, while steel reinforcement, post-tensioned tendons, and prestressed tendons are simulated using T3D2 truss elements. The connection between steel reinforcement and concrete is modeled with “embedded” constraints, and the interface between old and new concrete is treated as hard contacts. The interaction between the existing platform and replacement beam is simulated using tie constraints to maintain deformation coordination between the two. Secondly, Concrete materials are characterized using a plastic damage constitutive model, which not only captures the differences in tensile and compressive behaviors of concrete materials but also exhibits good convergence properties, making it suitable for describing the deformation characteristics of concrete materials under cyclic loading conditions. Thirdly, Steel reinforcement is represented using a bilinear elastic-plastic constitutive model. Furthermore, the measured values were used for the constitutive relations of the materials in the underpinning beam. To accurately reflect the fact that the underpinning nodes of the beam and original pile foundation bearing platform are different batches of poured concrete, this paper employs the contact surface to describe this feature. That is, the underpinning beam and the existing piers are defined as two surfaces, and then the contact relationship between the two surfaces is defined so that the contact surface becomes a kind of boundary condition, which is beneficial to the convergence of the calculation. According to the above modeling principles, the finite element model of a prestressed concrete underpinning node with interface contact between the old and new concrete is illustrated in Figure 10.

### 4.2. Comparison of Test and Finite Element Computation Results

**Figure 11:**
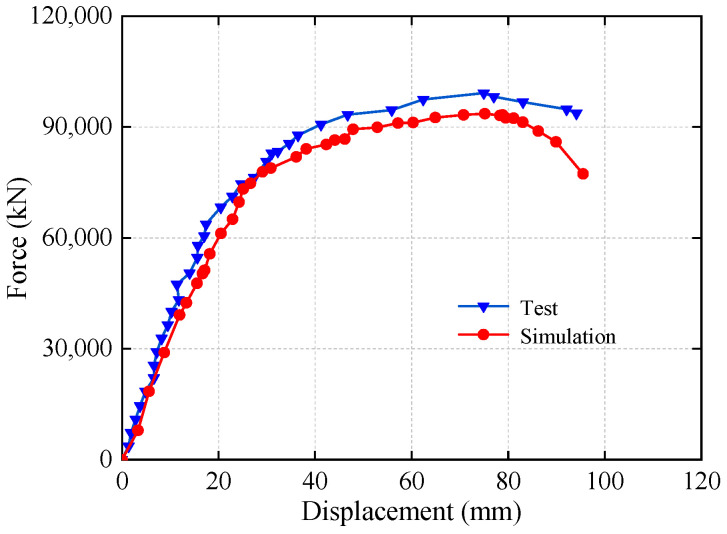
Test and finite element comparison.

This paper conducted a finite element simulation analysis of the underpinning structure and plotted the corresponding displacement curves under underpinning load in Figure 11 for easy comparison and analysis with the experimental results (Figure 8) in order to study the load-bearing performance of the underpinning structure with the new reinforcement technology.

As can be seen from Figure 11, the load–displacement curves of the test and simulation are in good agreement, and the ultimate loads of the two are, respectively 2750 kN and 2600 kN, with a 5.5% error, indicating that the scaled model of 1:6 is a good guide for the analysis of the prototype structure. The reason for the slight difference in displacement values between the above finite element results could be that the constitutive relationship of the material used in the finite element analysis differs from the actual values, resulting in the actual resistance of the structure being higher than the finite element analysis results. The elastic limit of the prototype structure in Figure 10 is approximately 68,400 kN, while the maximum load in the actual project construction process is 17,000 kN, indicating that the entire construction process is within the elastic stage of the underpinning beam structure and that this structure meets the basic bearing capacity requirements.

### 4.3. Displacement

**Figure 12:**
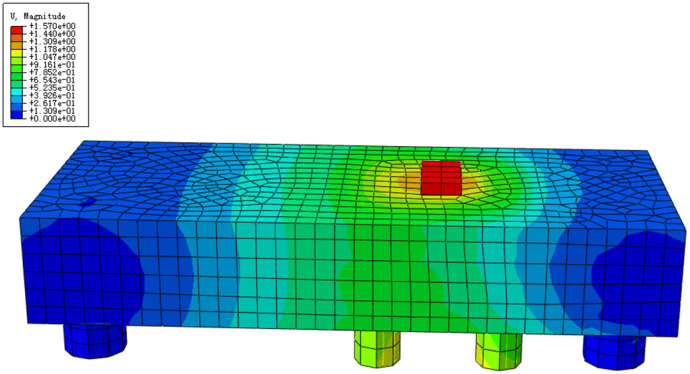
Displacement contour plot.

The displacement cloud of the prototype structure was acquired when the load was increased to the maximum value of 17,000 kN during the actual construction, as shown in Figure 12.

The maximum displacement of the underpinning beam structure is 15.7 mm, and the maximum deflection control value at the point of load is L/600, which calculates the allowable value of deflection for the structure to be 3.4 cm, which meets the specification requirements and ensures the smooth implementation of the actual project.

### 4.4. Underpinning Beam Structures Concrete Stress Analysis

**Figure 13:**
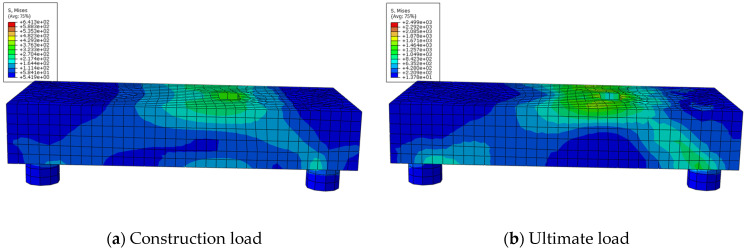
Stress distribution of concrete.

Figure 13 depicts the stress state of concrete under construction load and the ultimate load for underpinning beam structures. The underpinning beam concrete stress compression pattern in Figure 13 is in great agreement with the truss-arch model theory and in good accord with the theory presented in the literature [12]. As shown in Figure 13, when the load reaches its maximum value, the concrete strength is less than 6.41 MPa, and the entire concrete of the underpinning beam is in the elastic stage, indicating that the transfer of the underpinning load can be well realized throughout the construction process and that the underpinning beam still has sufficient safety reserves. When the load is increased to 90,000 kN, the underpinning beam’s local strength approaches the compressive strength of concrete, and it is generally between 10.49 MPa–16.71 MPa, indicating that the underpinning beam structure is still safe and trustworthy at this time.

### 4.5. Stress Analysis of Reinforcement in Underpinning Structure

**Figure 14:**
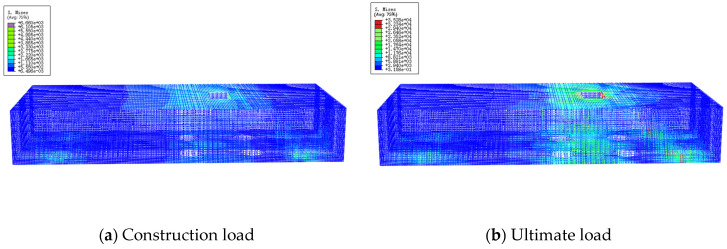
Stress of ordinary reinforcement.

**Figure 15:**
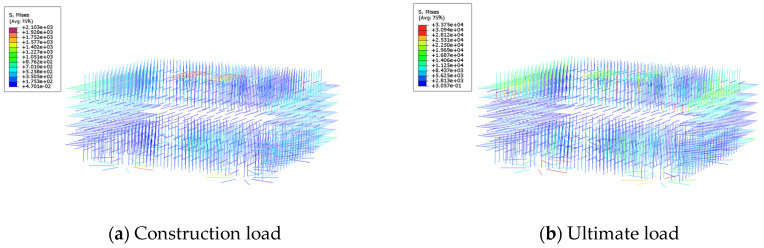
Stress of planting reinforcement.

**Figure 16:**
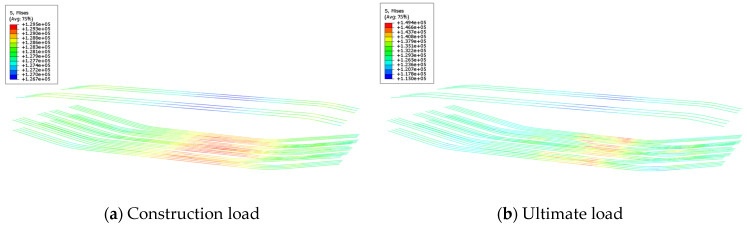
Stress of prestressed tendon.

Figure 14, Figure 15 and Figure 16 depict the stress analysis cloud for underpinning structure with conventional, planting, and prestressing reinforcement.

As shown in Figure 14, the stress of the reinforcement is less than 66.6 MPa under the action of the maximum construction load, and the reinforcement of the entire under-pinning beam is in the elastic stage, indicating that the reinforcement has a high safety reserve throughout the underpinning beam construction. When the load is increased to 90,000 kN, the underpinning beam’s local strength approaches the limit of tensile strength. However, the majority of reinforcement strength is maintained between 176.4–294 MPa, which is mostly in the elastic stage, and the tensile reinforcement corresponding to the bottom of the beam at the loading point has the largest tensile stress and has not yielded, indicating that the underpinning beam structure is still safe.

As shown in Figure 15, the planted reinforcement strength is under 21.3 MPa at the maximum construction load, and it is in the elastic stage, which indicates the planted bars still have enough strength reserve in the process of underpinning structure construction. When the load reaches 90,000 kN, only a small amount of reinforcement reach-es the tensile strength limit, while the majority of the other reinforcement strength is between 84.37–253.1 MPa, which is in the elastic stage, and the planted reinforcement transmits the load of the bearing platform to the underpinning beam structure.

From Figure 16, it can be seen that the prestressed tendons strength is within the range of 1274–1295 MPa under the maximum construction load, and the prestressed ten-dons stress is less than the tensile strength limit. Because of this, the prestressed ten-dons have sufficient safety reserves. When the load reaches 90,000 kN, prestressed ten-don stress is maintained between 1293 MPa and 1494 MPa, which is less than the tensile strength limit of 1860 MPa, and the prestressing tendon is still in a safe state under the ultimate load.

## 5. Conclusions

In this study, the displacements, stresses, and other properties of a pile foundation underpinning structure system in an urban overpass are explored using experimental and finite element analysis, and the following results are reached:

1. The damage condition of the pile foundation underpinning was slight under the test load, and there was no localized damage or general bending and shear damage, indicating the safety of “joints with rough interface + planting reinforcement + prestressing + epoxy resin reinforcing adhesive of planting rebar connection”.
2. The specimen deformation is small in the early loading stage, and the displacement curves of different measurement points are more coordinated, indicating that the combined surface of old and new concrete inside the underpinning beam body is still reliable. However, once the specimen enters the yielding stage, the deformation of the bearing platform section clearly shows an uncoordinated phenomenon, which indicates that the section of the beam is more concentrated in the force and that it is a weak region for the whole pile foundation beam structure system, and it should be noticed during the design.
3. The displacement cloud, reinforcement stress cloud, and concrete stress cloud of the pile foundation underpinning beam structure system show that the underpinning structure is in the elastic stage under the construction load, and even under the ultimate load, the underpinning beam structure does not show any overall damage, which indicates that the underpinning beam safety reserve is large enough.
4. The finite element simulation analysis of the overall model of the pile foundation beam structure shows that test results and simulation results are in great agreement, which verifies the accuracy of the modeling method and the test results. The finite element analysis results can be used as a supplement to the test, and then the detailed force conditions in the pile foundation beam structure system can be analyzed to guide the smooth implementation of the actual project.

## Figures and Tables

**Table 1:** Similar constants.

Type	Physical Quantities	Similarity Relation	Similar Constants
Material performance	Stress	S_σ_	1
	Strain	S_ε_	1
	Elastic modulus	S_σ_	1
	Poisson’s ratio	1	1
Geometrical performance	Geometrical size	S_l_	1/6
	Linear displacement	S_l_	1/6
	Angular displacement	1	1
Force	concentrated loads	S_l_^2^	1/36

**Table 2:** Geometrical properties of the model.

Items		Prototype (cm)	Model (cm)
Piers	Length	250	41.67
	Width	130	21.67
Abutment	Length	650	108.33
	Width	650	108.33
	Height	200	33.33
Underpinning beam	Length	2030	338
	Width	870	145
	Height	350	58.33

**Table 3:** Mechanical properties of steel and concrete.

Type	Physical Quantities	Yield Strength (Mpa)	Ultimate Tensile Strength (Mpa)	Extensibility
Reinforcement	Major reinforcement of the underpinning beam	396.33	548.33	6.5
	Other bars	346	463	6.1
	Prestressed tendons	--	1877	1.75
Concrete	Underpinning beam (The second pouring)	53.41		

**Table 4:** Material properties of epoxy resin anchoring adhesive for planting reinforcement.

	Thermal Distortion Temperature (°C)	Fracture Elongation (%)	Thermal Expansion Coefficient (10^−5^ m/(m·°C)	Tensile Strength (Mpa)	Compressive Strength (Mpa)
Epoxy	46–288	3–6	4.5–6.5	28–91	105–175

**Table 5:** Loading protocol.

	Preloading	Loading	
Loading protocol	0–100 kN	100 kN–1900 kN	1900 kN–2700 kN
	Loop loading twice	Loading step with 150 kN	Loading step with 100 kN
loading level	L0	L0–L12	L12–L20

## Data Availability

The data used to support the findings of this study are available from the corresponding author upon request.
